# Hyperspectral band selection using genetic algorithm and support vector machines for early identification of charcoal rot disease in soybean stems

**DOI:** 10.1186/s13007-018-0349-9

**Published:** 2018-10-03

**Authors:** Koushik Nagasubramanian, Sarah Jones, Soumik Sarkar, Asheesh K. Singh, Arti Singh, Baskar Ganapathysubramanian

**Affiliations:** 1 Department of Electrical and Computer Engineering, Iowa State University, Ames, IA USA; 2 Department of Agronomy, Iowa State University, Ames, IA USA; 3 Department of Mechanical Engineering, Iowa State University, Ames, IA USA; 4 Plant Sciences Institute, Iowa State University, Ames, IA USA

**Keywords:** Charcoal rot, Soybean disease, Precision agriculture, Band selection, Genetic algorithm, Support vector machines, Hyperspectral

## Abstract

**Background:**

Charcoal rot is a fungal disease that thrives in warm dry conditions and affects the yield of soybeans and other important agronomic crops worldwide. There is a need for robust, automatic and consistent early detection and quantification of disease symptoms which are important in breeding programs for the development of improved cultivars and in crop production for the implementation of disease control measures for yield protection. Current methods of plant disease phenotyping are predominantly visual and hence are slow and prone to human error and variation. There has been increasing interest in hyperspectral imaging applications for early detection of disease symptoms. However, the high dimensionality of hyperspectral data makes it very important to have an efficient analysis pipeline in place for the identification of disease so that effective crop management decisions can be made. The focus of this work is to determine the minimal number of most effective hyperspectral wavebands that can distinguish between healthy and diseased soybean stem specimens early on in the growing season for proper management of the disease. 111 hyperspectral data cubes representing healthy and infected stems were captured at 3, 6, 9, 12, and 15 days after inoculation. We utilized inoculated and control specimens from 4 different genotypes. Each hyperspectral image was captured at 240 different wavelengths in the range of 383–1032 nm. We formulated the identification of best waveband combination from 240 wavebands as an optimization problem. We used a combination of genetic algorithm as an optimizer and support vector machines as a classifier for the identification of maximally-effective waveband combination.

**Results:**

A binary classification between healthy and infected soybean stem samples using the selected six waveband combination (475.56, 548.91, 652.14, 516.31, 720.05, 915.64 nm) obtained a classification accuracy of 97% for the infected class. Furthermore, we achieved a classification accuracy of 90.91% for test samples from 3 days after inoculation using the selected six waveband combination.

**Conclusions:**

The results demonstrated that these carefully-chosen wavebands are more informative than RGB images alone and enable early identification of charcoal rot infection in soybean. The selected wavebands could be used in a multispectral camera for remote identification of charcoal rot infection in soybean.

## Background

Soybean [*Glycine max* (L.) Merr.] is the major oilseed crop grown in the United States [1]. Soybean is also economically important as it is the second major crop overall produced by the United States [1]. Soybean is used to produce biofuel, cooking oil, soy foods, and animal feed, among many other uses, but the crop is threatened by over 100 diseases with 35 believed to be important pathogens affecting soybean yield [2, 3].

Charcoal rot is an economically critical disease that affects soybean, as well as 500 other plant species worldwide, and is caused by the fungal pathogen *Macrophomina phaseolina* (Tassi) Goid [4–6]. Infection is favored by warm (30–35 °C), dry, drought-like conditions but can cause up to 50% yield loss even in irrigated environments [7–10]. Charcoal rot earned its common name from the gray-silver discoloration caused by microsclerotia formation in the vascular tissue and pith of lower stems and roots of infected plants [7, 11]. These microsclerotia are small dark survival structures that persist in the soil and plant debris after harvest and can act as an inoculum source for charcoal rot infection during the next growing season [3, 7, 12]. Symptoms generally become visible at the R5–R7 reproductive stages, or from early seed to early maturity, but can occasionally be seen earlier as reddish-brown lesions on the hypocotyl of seedlings [3, 7]. In more mature infected plants, a reddish-brown discoloration of the vascular tissue in the roots and lower stem generally precedes foliar symptom development [7]. Following internal discoloration, diseased plants may yellow, then wilt, and prematurely senesce leaving dead leaves and petioles still attached to the stem [3, 7, 13]. Black microsclerotia on the above ground plant are first visible at the stem nodes and can be seen in the epidermal and sub epidermal tissue of plant stems as well as scattered on dry pods and seed of more mature plants [3, 7]. Management of charcoal rot has proven to be difficult as no fungicides are available for control and more work needs to be done to research the potential of seed treatments [3, 12]. In addition, crop rotation may not be a viable strategy to manage infection, because charcoal rot infects the United States’ major crops including corn, cotton, and sorghum [14, 15]. Furthermore, no commercial soybean varieties are considered resistant, though a few varieties demonstrate moderate resistance [8, 13, 16–19]. However, a genome wide association (GWA) study across both field and greenhouse environments recently reported a total of 19 single nucleotide polymorphisms (SNPs) associated with charcoal rot resistance in soybean [20]. While over 800 soybean lines have been evaluated for charcoal rot resistance, identification of resistant genotypes has been limited due to a *need for an accurate, rapid, and consistent method for disease assessment and classification* [12, 13].

### Current state of disease assessment and outlook

Multiple methods, which are predominantly visual, have been proposed for assessing charcoal rot severity of soybean plant canopies, roots, and stems in the field and indoor environments. These methods include evaluation of the intensity or length of stem and root discoloration caused by microsclerotia formation, evaluation of the percent chlorosis and necrosis of the plant canopy throughout the growing season, chlorosis and necrosis of foliage that remains attached to the plant at R7, calculation of colony forming unit index to quantify the microsclerotia content in the stem and root, and lesion length measurements of cut-stem inoculations on young plants [13, 19, 21, 22]. However, visual rating methods can be subjective and are susceptible to human error caused by rater ability, and inter/intra-rater reliability [23–28].

Furthermore, visual ratings only take advantage of visible wavelengths of the electromagnetic spectrum [23]. Hyperspectral imaging can capture both spectral and spatial information from a wider range of the electromagnetic spectrum including the visible and near-infrared regions [29]. Automating disease severity rating through hyperspectral imaging offers a potential solution to the standardization and reliability issues in current visual rating systems. While some hyperspectral systems do not incorporate imaging, but rather average all spectra obtained from a given area, the imaging aspect inherent in hyperspectral imaging techniques comparing to non-imaging hyperspectral systems offers many benefits for studying plant disease symptoms [30]. Extraction of reflectance spectra from each pixel, enables one to relate changes in reflectance values to disease symptoms [31, 32]. Recent plant pathology and phenotyping studies have utilized hyperspectral imaging data to study the effect of different plant pathogens. Examples include approaches to identify differences in the reflectance patterns of resistant and susceptible barley genotypes inoculated with powdery mildew [30, 33] the content of charcoal rot (*M. phaseolina*) microsclerotia in ground root and stem tissue as a method for rating infection severity [34], and hyperspectral imaging to distinguish between the symptoms of Cercospora leaf spot, powdery mildew, and leaf rust at different developmental stages in sugar beet [32].

A key issue with utilizing hyperspectral imaging is that the resulting hyperspectral data cubes, or the 3-dimensional output of hyperspectral imaging comprised of 2 spatial dimensions and 1 wavelength dimension, are high dimensional and contain redundant information which reduces the ability to distinguish between different object classes in classification problem. [35]. Using a hyperspectral camera on a drone for crop disease identification and phenotyping can also generate large quantities of data during the flight making it necessary to have a large on-board storage capacity and also substantially increases computational cost for any subsequent analysis. Therefore, there is a need to develop an analysis pipeline to reduce dimensionality of the data and to select optimal wavelengths that are most useful for phenotyping and disease identification. This serves as the motivation of this study.

Feature extraction and feature selection are two different methods for dimensionality reduction of hyperspectral data. Feature extraction methods such as Principal Component Analysis (PCA), Linear Discriminant Analysis (LDA), Independent Component Analysis (ICA) and Maximum Noise Fraction (MNF) project the original hyperspectral data into a new low-dimensional data by reducing the spectral dimension [36–39]. Feature extraction methods alter the physical meaning of the hyperspectral data during transformation to a new (and lower) dimensional space whereas feature selection methods preserve the original features [40]. Feature selection essentially boils down to carefully selecting a subset of the available wavebands (i.e. waveband selection) that preserves certain traits of the full dataset [41]. Feature selection methods are broadly classified into supervised or unsupervised methods [42]. Supervised methods use input and desired output variables for training an algorithm whereas unsupervised methods use only the input data for training [43]. Some supervised waveband selection methods use class separability metrics like Euclidean distance, transformed divergence, Bhattacharyya distance, Jeffreys–Matusita (JM) distance [44, 45]. A waveband selection method based on estimation of mutual information for classification of hyperspectral images was proposed by Guo et al. [46]. Sequential search strategies like Sequential Forward Selection (SFS), Sequential Floating Forward Selection (SFSS), Sequential Backward Selection (SBS) and Sequential Backward Floating selection (SBSS) have also been used for waveband selection [47, 48]. These sequential search algorithms are simple and suboptimal. Evolutionary methods such as Particle Swarm Optimization (PSO) and genetic algorithms (GA) which can search for global optimal solutions have been found to be successful in effective waveband selection [49, 50]. In this study, we use an evolutionary method, specifically GA, as an optimizer along with Support Vector Machine (SVM) [51] as a classifier for effective waveband selection. GA-SVM based model have been successful in waveband selection for classification of remotely sensed hyperspectral images [49, 52–55]. Although computationally costly, evolutionary algorithms can give better optimal solution than sequential algorithms since the best feature combination is selected simultaneously [56].

The objectives of this study were (1) hyperspectral imaging enabled early identification of charcoal rot disease and (2) to determine the most effective minimum number of wavebands for discrimination of healthy and charcoal rot infected stems. This study shows that a genetic algorithm-support vector machine based model can be used in selecting the most effective waveband combination for early detection of charcoal rot disease in soybeans. Additionally, using F1-Score as an optimization metric instead of classification accuracy can overcome the skewness of classification accuracy metric for the dominant class of an imbalanced dataset (number of healthy samples more than the number of infected samples) [57].

## Methods

### Plant material

Four soybean genotypes, Pharaoh (susceptible), PI479719 (susceptible), DT97-4290 (moderately resistant), and PI189958 (moderately resistant) were included in this study. Two seed of each genotype were planted in a commercial soil substrate (Sungro horticulture professional growing mix) in 8 oz styrofoam cups in a growth chamber at 30 °C day/21 °C night with a 16-h photoperiod. Each styrofoam cup was supplemented with 1/8tsp (0.65 g) of osmocote 15-9-12 at planting. Ten days after planting, plants were thinned down to one plant per pot choosing the most vigorous plant. Plants were arranged in a randomized complete block design with four replications. The two treatments were inoculation and mock-inoculation. Data collection was completed within 15 days after inoculation (DAI). Replication 1 was planted in the growth chamber in September 2016. Lesion lengths and hyperspectral images were collected at 3, 6, 12, and 15 DAI to study the earlier and then later time points post inoculation. Replications 2–4 were planted together in November 2016. Lesion length ratings and data cubes were collected at 3, 6 and 9DAI in replications 2–4 focusing on the earlier disease development time points.

### Culture and inoculation of *M. phaseolina*

The pathogen *M. phaseolina* 2013X, originally collected from the field in Iowa in 2013, was re-isolated from inoculated stems of soybean plants grown in the growth chamber. Inoculation was performed 3 weeks (21 days) after planting of seeds. In order to prepare for inoculation, cultures of *M. phaseolina* were started in the lab, 17 days after planting (i.e. 4 days before inoculation). This culture preparation consisted of transferring 0.5 cm plugs of *M. phaseolina* to Potato Dextrose Agar (PDA) plates which were then stored in the dark at 30 °C for 4 days. Twenty-one days after planting, the four soybean genotypes were inoculated according to the cut-stem inoculation technique [22]. Sterile 200 µl pipette tips were placed open end down into the media around the leading edge of the fungal colony cutting a small disk of media and fungal hyphae from the plate. Each soybean stem was severed exactly 40 mm above the unifoliate node. A pipette tip was removed from the culture plate ensuring that it carried a disk of PDA media + *M. phaseolina* mycelia for the inoculation treatment. The pipette tip was pushed onto the cut stem, like a hat, and the open wound imbedded in the media. The same protocol was carried out for the mock-inoculation treatment using uncontaminated plates of PDA media. Three days after inoculation, pipette tips were removed from all plants.

### Hyperspectral image acquisition

Pika XC hyperspectral line scanning imager (Resonon, Bozeman, MT) was used to construct hyperspectral data cubes of soybean stems. The Pika XC imager has a spectral resolution of 2.5 nm, with 240 spectral channels covering a spectral range from 382 to 1032 nm. Hyperspectral images of healthy and charcoal rot infected stems were collected at different time points, as explained previously, for classification.

The imaging system also includes a mounting tower, linear translation stage, and a computer pre-loaded with SpectrononPro software (Resonon, Bozeman, MT). Illumination was provided by two 70-watt quartz-tungsten-halogen Illuminator lamps (ASD Inc., Boulder, CO) which provide stable illumination over a 350–2500 nm range. The distance between the lamps and the plant stem being imaged was 54 cm with lights pointed towards the sample at a 45-degree angle. Prior to imaging, the ASD pro-lamps were turned on and warmed up for at least 20 min to produce a stable light source.

**Fig. 1:**
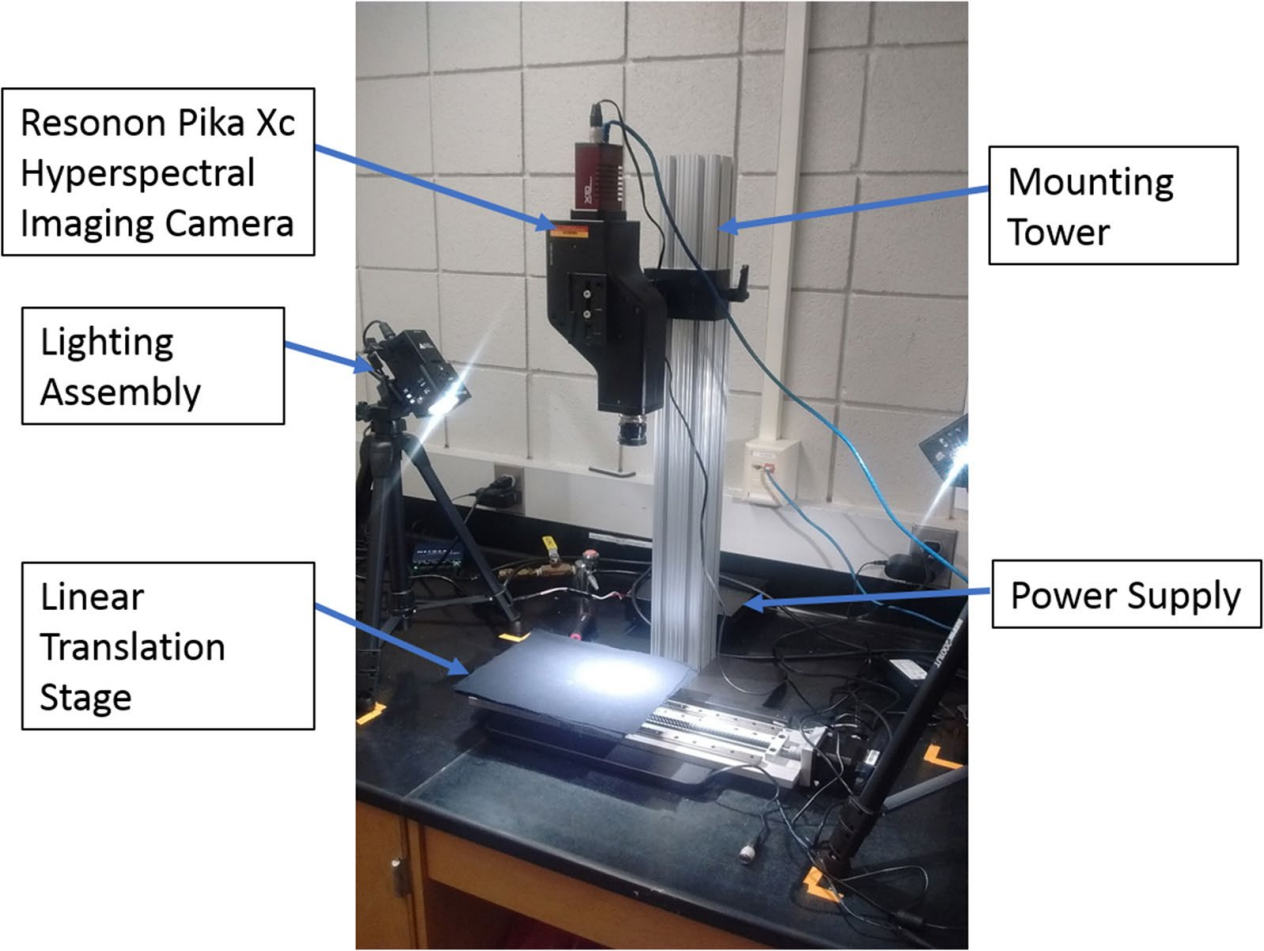
Illustration of the hyperspectral imaging setup for charcoal rot disease detection

Using the SpectrononPro software interface, the camera exposure was set automatically, and focus adjusted manually using a lens of f-number (ratio of focal length and diameter of a lens) of ƒ/1.4. The system was then calibrated to a white reference tile and a dark reference with the lens cap covering the objective lens. Aspect ratio was adjusted using a concentric circles sheet provided by Resonon. Data was captured with reflectance values between 0 and 1. Figure 1 shows the hyperspectral imaging setup used in the study. The specimen was placed horizontally in the linear translator stage with the lesion on the right side.

Plant stems were destructively imaged at different time points after inoculation (3, 6, 9, 12 and 15 DAI). All leaves were removed from the plant stem and the stem severed at the soil surface immediately prior to hyperspectral data cube collection. Stems were placed on the linear translation stage for imaging. Growth patterns of stem lesions often resulted in irregular lesion boundaries. So, stems were positioned on the linear translation stage so that the longest edge of the lesion was facing the camera lens. Following calibration, a data cube was collected from each stem. The hyperspectral data cubes and corresponding RGB images were saved on an external hard drive.

### Charcoal rot rating protocol

**Fig. 2:**
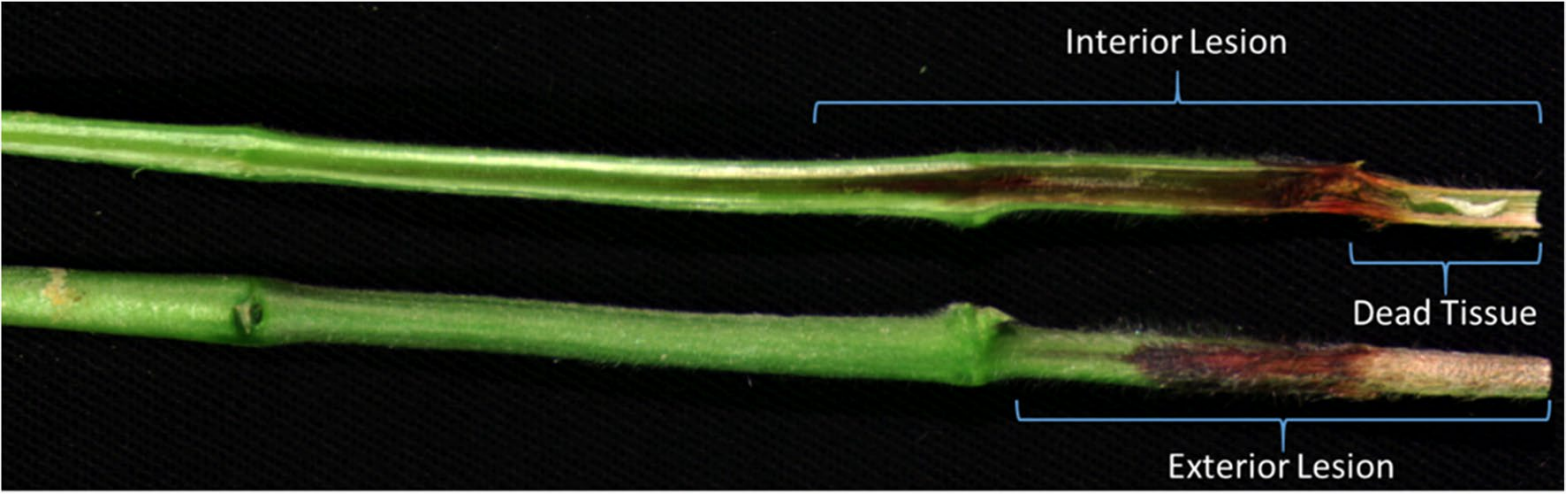
Charcoal rot disease ratings were obtained by measuring three different lesion elements of symptom development including the exterior lesion, dead tissue, and interior lesion length (mm)

In addition to stem images, disease progression was manually rated by measuring length (mm) of the exterior lesion, interior lesion, and dead tissue lesion. The exterior lesion was clearly visible as a reddish-brown to black discoloration proceeding from the inoculated end of the stem. The interior lesion, a reddish-brown discoloration of the vascular tissue, progressed farther than the exterior reddish-brown lesion and was measured to the lowest point of the dark reddish continuous discoloration from the inoculated end of the stem. Tissue death was the last symptom to develop and as such, the dead tissue lesion was shorter than the interior and exterior lesions and was measured to the extent of the dry, dead plant tissue. Measurement protocol was designed based on Twizeyimana et al., where charcoal rot lesion length was measured from the unifoliate node to the lowest edge of the lesion being measured [22]. Figure 2 shows the interior and exterior and dead tissue lesion lengths of an infected soybean stem.

### Genetic algorithm-support vector machine based feature selection

#### Problem definition

**Fig. 3:**
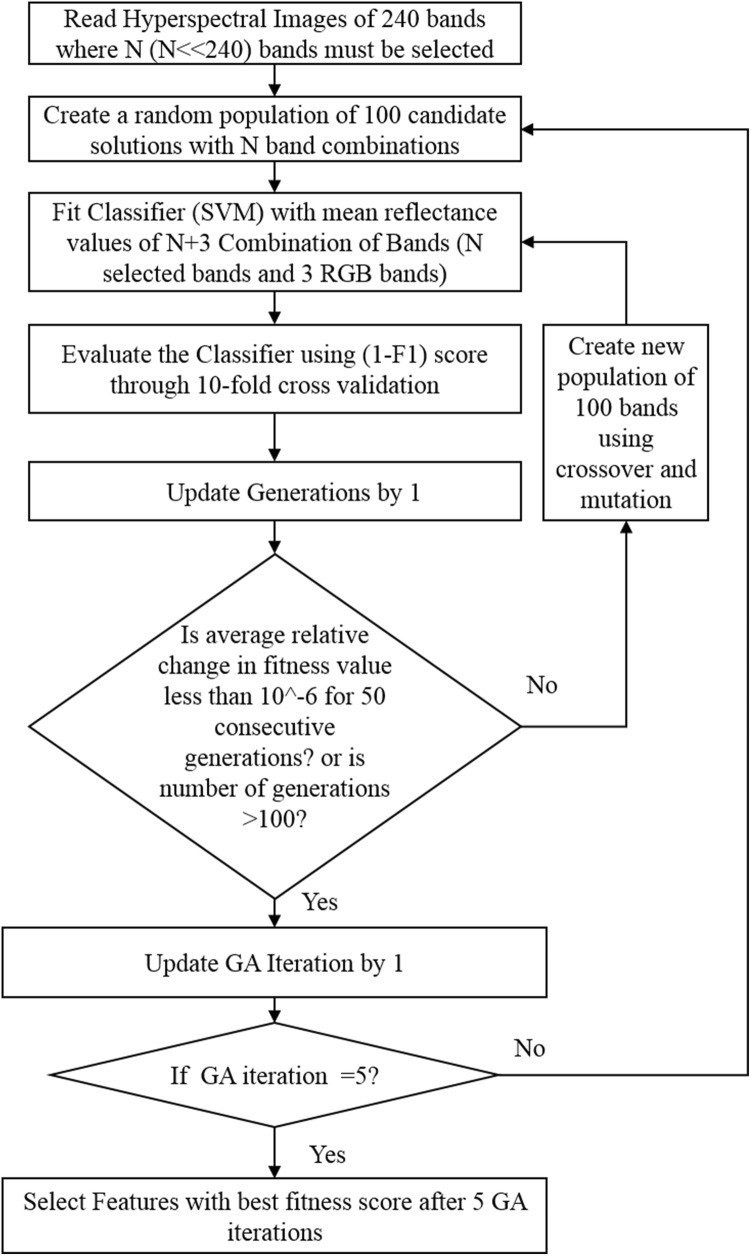
GA-SVM architecture for selection of optimal bands

The identification of best waveband combination for maximally discriminating healthy and charcoal rot infected stems from a set of 240 wavebands was formulated as an optimization problem. A genetic algorithm (GA) based optimization protocol using support vector machine (SVM) as a classifier was used to find the most optimal wavebands for designing a multispectral camera system for phenotyping and disease identification. Spectral and spatial information from the hyperspectral images were used for early identification and classification of disease. The objective of the optimization was to find the best waveband combination that maximizes the classification performance (i.e. find the best k waveband combination that produces the best classification performance when distinguishing between healthy and diseased specimens). Figure 3 shows the flowchart of the GA-SVM architecture for waveband selection. MATLAB R2017a was used to implement the GA-SVM model.

#### Support vector machine

Support Vector Machine (SVM) is a kernel-based discriminative supervised learning algorithm for classification [51, 58]. SVM is one approach for constructing a classifier that maps an input data (of N waveband information) to a class (healthy vs infected). SVM has been used with significant success in identification of variety of plant stresses [43, 59]. Formally, SVM projects data which are not separable linearly into a higher dimensional space using a kernel and separates the classes with an optimal hyperplane that maximizes the margin between the classes [60]. In this study, we used Radial Basis Function (RBF) [61] kernel to learn the non-linear classifier. SVM has been used as a classifier in wrapper based feature selection methods for classification of hyperspectral images [49, 52, 54, 55, 62–65]. After trial and error, the two Radial Basis Function (RBF) kernel parameters C and γ were set to 1000 and 1, respectively.

#### Genetic algorithm

Genetic algorithms are population based stochastic search optimization techniques inspired by natural selection and natural genetics principles [66]. The population of candidate solutions (i.e. wavebands) is represented as a long string of bits and is called ‘chromosome’. Each of these chromosomes is assigned a score using a fitness function for evaluation [67]. In this case, the fitness function evaluates how well the chromosome (i.e. that particular selection of wavebands) performs to distinguish between diseased and healthy specimens. These chromosomes are evolved in successive generations using selection, mutation and crossover genetic operators for exploring the solution space until a best solution is obtained, or termination criteria is encountered. Selection of chromosomes for reproduction can be done in diverse ways [68]. One of the ways is to choose the pair of chromosomes in the population that provides relatively good fitness scores to perform crossover. Crossover operator randomly combines genetic information of two chromosomes. Mutation operator modifies some component of a chromosome to form random new populations in the search space which prevents GA from choosing local optimal solutions. The “elite” is a GA hyperparameter decides the number of most-fit individuals passed from one generation to the next generation without changing. This process of selection, mutation and crossover is repeated for multiple generations to improve the population fitness [66] (Fig. 3).

It is important to carefully choose a well-defined and appropriate fitness function. After exhaustive numerical tests and exploration, we chose the F1 score of the infected class as a useful tool to evaluate performance of the classifier. F1-score (Eq. 3) of the infected class have been used previously for evaluating plant disease classifiers [69, 70]. Maximizing only precision (Eq. 1) or recall (Eq. 2) does not imply good classification performance [71]. F1 score is defined as harmonic mean of precision and recall values providing equal weightages to both precision and recall scores [72]. A good F1 score is also indicative of good classification performance. Equations 1, 2, and 3 provide the formulas for precision, recall, and F1 score metrics where TP is True Positive, FP is False Positive, and FN is False Negative. The value of F1 score can vary from 0 to 1. A value of 1 and 0 is obtained for best and worst classification performance respectively. We conduct a 10-fold cross-validation on the complete training data for evaluation of the SVM classifier. The mean value of the 10 F1-scores from the 10-fold cross-validation was used as a fitness value for the GA. F1 score is a better metric over classification accuracy for measuring the classification performance of an imbalanced data, as classification accuracy is a biased metric which favors the class with more samples (healthy samples in our case) [57]. The objective of the GA was to find the best waveband combination that maximizes the F1 score. Table 1 shows the variables of the confusion matrix to analyze the performance of the classification.

1 $$ Precision = \frac{TP}{TP + FP} $$

2 $$ Recall = \frac{TP}{TP + F N} $$

3 $$ F1\,Score = \frac{2*Precision*Recall}{{\left( {Precision + Recall} \right)}} $$

**Table 1:** Confusion matrix definition

	Infected (Predicted)	Healthy (Predicted)
Infected (Actual)	True Positive (TP)	False Negative (FN)
Healthy (Actual)	False Positive (FP)	True Negative (TN)

The termination criteria depend on the average change in fitness value for 50 continuous generations or the maximum number of generations allowed which were 100 in our study. The last generation of GA iteration will contain the most optimal solution.

We choose to augment the hyperspectral wavebands with some visible spectrum (RGB information). We do this since RGB cameras are inexpensive, light weight, and can be attached to drones easily for capturing images. Therefore, the input feature to the SVM classifier consists of a fixed part and variable part. The mean values of reflectance from three wavelengths 475.56 nm, 548.91 nm and 652.14 nm representing red, green and blue colors respectively were used as fixed part of the input feature. The variable part of the input feature was chosen by the GA. The input chromosome comprises of bits each representing one of the total 240 wavebands of the input hyperspectral image. The number of bits in a chromosome is equal to the total number of wavebands to be selected by the GA. The number of bits chosen were 3 in our study. In total, the input features consisted of six wavelengths, including RGB and the wavelengths selected by the GA. Binary tournament [68], Laplace [73], and power methods [74] were used for selection, crossover and mutation respectively. Table 2 provides the implementation details of the GA.

**Table 2:** Implementation details of genetic algorithm

Parameters	
Number of genetic algorithm iterations	5
Population	100
Maximum number of generations	100
Crossover probability	0.8
Elite count	2
Mutation probability	0.2
Selection	Binary selection tournament
Crossover	Laplace crossover
Mutation	Power mutation
Stopping criteria	Average change in best fitness value is less than 10^−6^ for 50 generations or number of generations = 100

#### Data pre-processing

The dataset contains 111 hyperspectral images of size 500 × 1600 × 240 pixels. Replications 1–4 provided 39, 24, 24, and 24 data cubes respectively. Data cubes from each replication were distributed among the training and testing datasets. Seventy-two hyperspectral images were used for training and 39 hyperspectral images were used for testing. The training set had 35 data cubes of healthy stems and 37 data cubes of diseased stems. The testing set had 21 data cubes of healthy stems and 18 data cubes of diseased stems. Since the number of test data was small, to increase the amount of data for developing the model and for prediction of disease progression to get a better understanding of severity of the disease spread, each of the hyperspectral stem images was divided into patches of size 500 × 64 × 240 pixels for training and testing purpose [75] (Fig. 5). The healthy (mock-inoculated) and diseased (inoculated) samples allowed for testing and training for classification of diseased compared to healthy tissue. Training data was labeled using ground truth data of the measured interior lesion length (mm). A summary of the ground truth data for interior lesion length as well as the exterior and dead tissue lesion lengths can be seen in Table 3. The interior lesion length, measured in mm on the interior of the stem, was used for ground truth labelling of the image patches. Time points 3 and 6 each contain 4 replications while time point 9 contains 3 replications. The decrease in sample numbers in 3 DAI interior and dead lesions lengths as well as 9 DAI exterior lesion length are a result of missing data points caused during data transfer. A stem is determined as infected if at least one of the image patches of the stem is predicted as infected.

**Table 3:** Mean and standard error of the mean for lesion length

Trait	Time point	Genotype	Number of samples	Mean (mm)	Standard error mean
Exterior lesion length	3 DAI	DT97-4290	4	31.5	8.5
		Pharoah	4	28.0	4.7
		PI189958	4	25.5	4.5
		PI479719	4	18.0	3.7
	6 DAI	DT97-4290	4	31.0	7.1
		Pharoah	4	28.5	4.4
		PI189958	4	28.5	2.5
		PI479719	4	22.8	2.3
	9 DAI	DT97-4290	3	34.3	6.2
		Pharoah	3	39.7	5.8
		PI189958	2	20.0	1.0
		PI479719	3	36.0	4.0
Interior lesion length	3 DAI	DT97-4290	4	29.0	7.0
		Pharoah	4	35.0	2.1
		PI189958	4	30.0	3.0
		PI479719	3	46.0	9.6
	6 DAI	DT97-4290	4	37.5	6.3
		Pharoah	4	49.8	9.5
		PI189958	4	34.3	3.6
		PI479719	4	26.5	6.8
	9 DAI	DT97-4290	3	68.3	12.3
		Pharoah	3	61.0	10.7
		PI189958	3	41.0	2.5
		PI479719	3	66.3	12.4
Dead lesion length	3 DAI	DT97-4290	4	17.3	6.6
		Pharoah	4	20.3	5.5
		PI189958	4	18.3	2.5
		PI479719	3	23.3	0.9
	6 DAI	DT97-4290	4	25.0	6.4
		Pharoah	4	22.8	5.0
		PI189958	4	16.0	1.8
		PI479719	4	16.8	3.0
	9 DAI	DT97-4290	3	32.3	5.7
		Pharoah	3	32.3	4.9
		PI189958	3	12.0	4.6
		PI479719	3	28.7	5.2

The lesion length measurements are from the three earliest time points of lesion rating [3, 6 and 9 days after inoculation (DAI)]. Due to the destructive nature of data collection individual lesion progression could not be tracked past the date of imaging. Because of the destructive nature as well as variability in samples, and the expected trend of lesion length increasing over time is not always observed

## Results and discussion

### Spectral reflectance

**Fig. 4:**
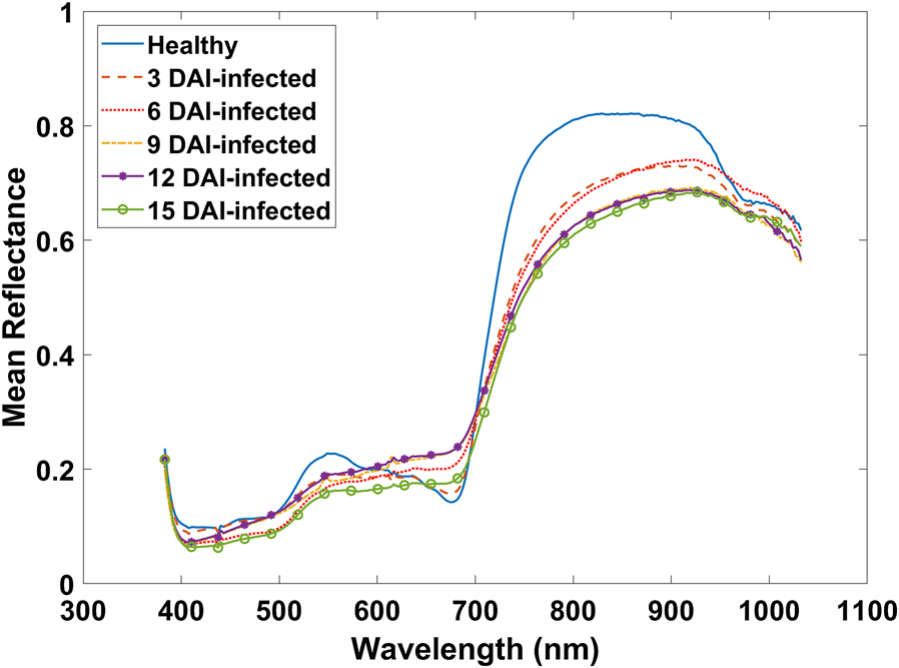
Mean spectral reflectance curves of healthy and infected stems

Figure 4 shows an example of mean reflectance curves of healthy and infected samples at various stages. The mean reflectance value of a wavelength is obtained by spatially (500 × 1600) averaging the reflectance values in that wavelength. It is seen that the maximum reflectance value of infected samples is less than the healthy sample and the trends of all infected samples looks similar. The reflectance value decreases as the severity of the charcoal rot disease increases.

### Feature selection

The number of wavebands used for classification were reduced from 240 to 6 using our GA-SVM model. 475.56(B), 548.91(G), 652.14(R), 516.31, 720.05, 915.64 (wavelengths in nm) are the maximally effective 6 waveband combinations selected by the GA-SVM model including RGB wavebands. The confusion matrices for the RGB wavelengths and selected wavelength combination are shown in Table 4. Table 5 shows the comparison of binary classification for the RGB and selected wavelengths. The F1 score of the infected class and overall classification accuracy were 0.769 and 76.92% respectively using only RGB wavelengths whereas classification accuracy of 97% and F1-score of 0.97 for 39 test stems were obtained using the selected 6 waveband combinations of GA.

**Table 4:** Confusion matrix of test samples from 3, 6, 9, 12 and 15 DAI

Waveband combination	Confusion matrix	
3 (RGB)	TP = 17	FP = 8
	FN = 1	TN = 13
6	TP = 18	FP = 1
	FN = 0	TN = 20

**Table 5:** Classification results of test samples from 3, 6, 9, 12 and 15 DAI

Waveband combination	Precision	Recall	F1-score	Healthy**	Infected**	Overall accuracy (%)
3 (RGB)	0.68	0.94	0.79	92.85	68	76.92
6	0.94	1	0.97	100	94	97

**Per class accuracy (%)

The RGB wavelengths alone did not perform well, which might be because of their inability to differentiate between the reflectance values of a healthy stem and charcoal rot infected stem. The classification accuracy and F1 score of the selected 6 waveband combinations indicate that they were good at distinguishing between healthy and charcoal rot infected samples.

### Early disease detection for 3-DAI samples

The ability to detect disease early is very important for mitigation. Among 39 test stems, 11 were collected at 3-DAI. Out of 11, 6 represent healthy stems and 5 were infected. The binary classification results for 3-DAI samples are shown in Table 6. The classification accuracy and F1-score were 81.82% and 0.83 respectively using RGB wavelengths whereas the classification accuracy and F1 score were 90.91 and 0.90 respectively using the 6 waveband combinations. These results indicate that the specific wavelengths chosen in the six waveband combinations are responsive to disease symptoms even at the early stage of infections.

**Table 6:** Classification results for 3-DAI samples

Waveband combination	Confusion matrix		Precision	Recall	F1	Healthy**	Infected**	Overall accuracy (%)
3(RGB)	TP = 5	FP = 2	0.71	1	0.83	100	71.43	81.82
	FN = 0	TN = 4						
6	TP = 5	FP = 1	0.83	1	0.90	100	83.33	90.91
	FN = 0	TN = 5						

**Per class accuracy (%)

### Disease length prediction

**Fig. 5:**
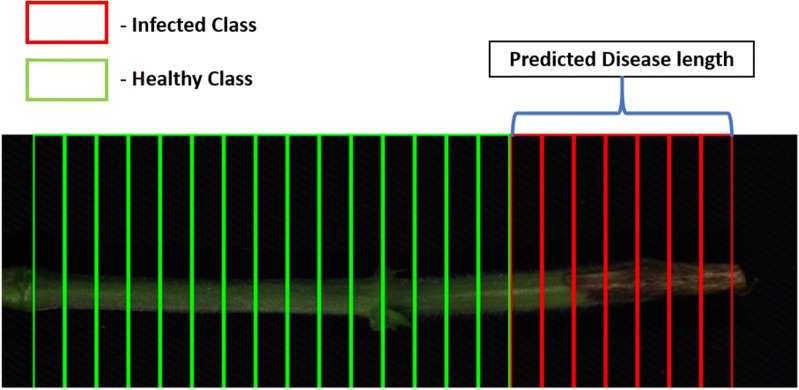
Prediction of stem patches by selected optimal wavelengths

Identification of charcoal disease length progression is important for understanding the severity of the disease and helpful in understanding the resistance of various soybean genotypes to the disease. Figure 5 shows the predictions for each patch in an inoculated stem.

**Fig. 6:**
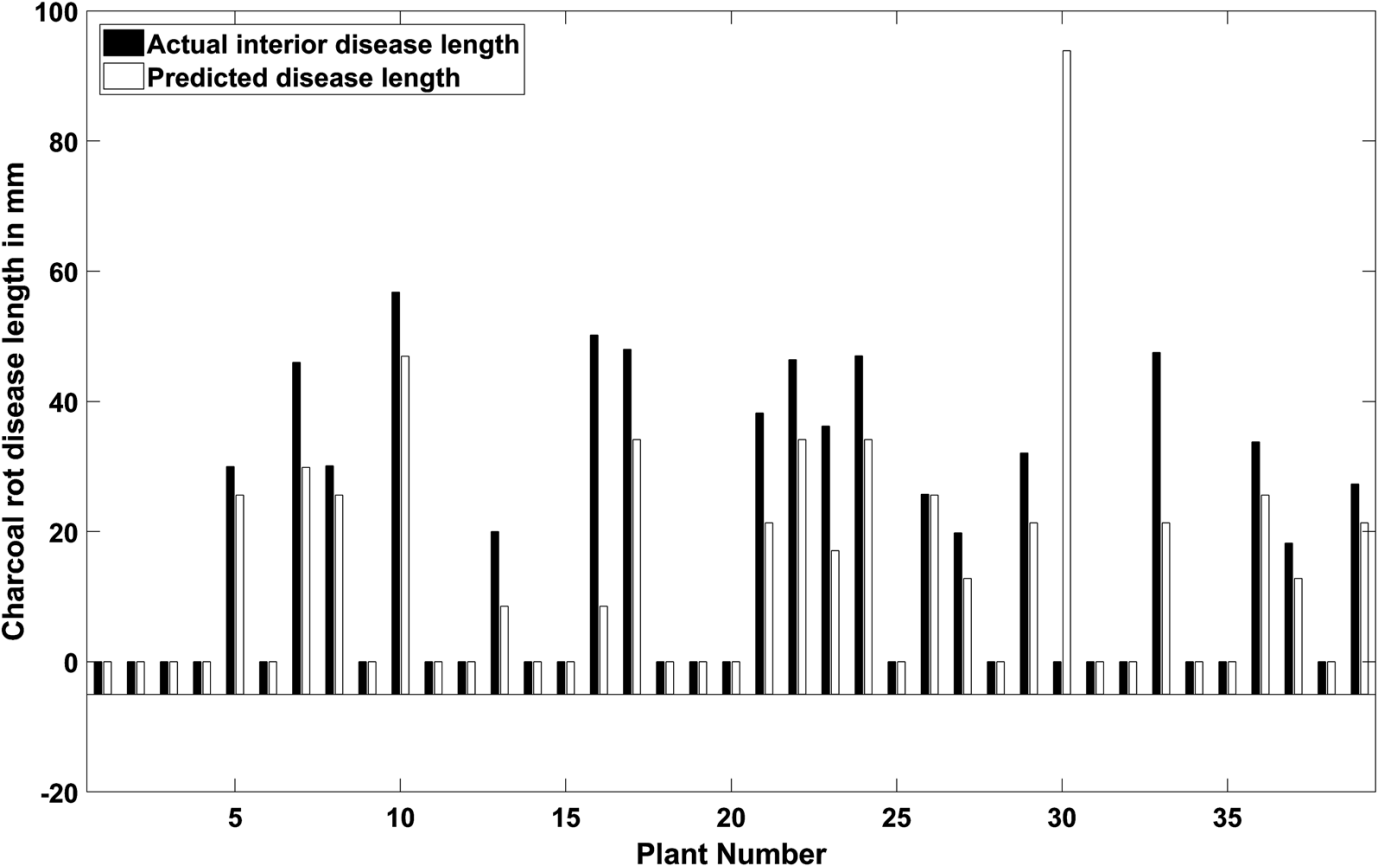
Actual disease progression length (mm) compared to predicted disease progression length based on patch wise classification results

The total disease length is the distance from the inoculation point to the end of the farthest patch which was predicted as infected from the inoculation point. The total predicted disease length could be calculated by summing the length of the number of patches in one stem data cube classified as diseased. The predicted disease lengths for 39 test stems are shown in Fig. 6. The disease length prediction for stem number 30 was incorrect due to misclassification of a patch at the end of the stem. For other stem samples, the predicted disease lengths were equal or proportional to the interior lesion length.

## Conclusions

Hyperspectral images of four different soybean genotypes (two susceptible and two moderately resistant), half healthy and half infected with charcoal rot disease were collected at 5 different time points post infection. The main objectives of this study were to identify the most effective minimal number of wavebands from a set of 240 hyperspectral wavebands that are required for identification of charcoal rot disease and to analyze the performance of these wavebands in early detection of the disease.

The study used both spectral and spatial information (mean value of reflectance from different wavelengths) for disease identification. Due to imbalanced dataset of healthy and infected stems used in our study, the SVM classification performance which was optimized using GA for optimal waveband selection was evaluated for maximizing the F1 score value of the infected class instead of overall classification accuracy.

An effective six waveband combination for discrimination of healthy and charcoal rot infected stems was found. Early identification of charcoal rot disease at 3 days after inoculation was possible using the selected waveband combinations. The GA-SVM model obtained F1-score of 0.97 and classification accuracy of 97% using selected 6 hyperspectral waveband combinations for complete test data (samples from 3, 6, 9, 12 and 15 DAI). These results were 26.1% better than those obtained using only the visible RGB wavelengths highlighting the importance of including the additional non-visible wavelengths for disease detection. The F1-score and classification accuracy for early detection (3-DAI samples) samples were 0.90 and 90.91% respectively using the selected 6 wavebands. Two out of the three wavelengths selected (720.05 nm, 915.64 nm) along with the RGB wavebands in the six waveband combinations were selected in the near-infrared region and one was selected in the visible region (516.31 nm) indicating that both near infrared region and visible region were useful in early identification of charcoal rot disease. This relationship between the stem reflectances and charcoal rot disease is along the lines of the results of a previous study [34]. Genotypes with susceptible and moderately resistant responses to charcoal rot were used in this study. The length of disease progression (mm) in each stem was measured to understand the severity of the disease spread among different genotypes. Using hyperspectral imaging combined with GA-SVM enabled waveband selection resulting in a higher classification accuracy compared to visible wavelengths alone. However, this study focused on indoor imaging so future work should utilize field inoculations and evaluations to expand this technology into the field. Furthermore, field inoculations of diverse soybean genotypes will be imaged using a multispectral camera with the selected wavebands from the GA-SVM model for early identification of charcoal rot disease to understand the disease resistance of specific genotypes. Also, the length of disease progression in different genotypes will be studied with larger sample size to characterize their disease resistance. In conclusion, this study provides an efficient methodology for selecting the most effective wavebands from hyperspectral data to be used for early disease detection of charcoal rot in soybean stems.
